# Severe *Plasmodium knowlesi* infection with multi-organ failure imported to Germany from Thailand/Myanmar

**DOI:** 10.1186/1475-2875-13-422

**Published:** 2014-11-04

**Authors:** Michael Seilmaier, Wulf Hartmann, Marcus Beissner, Thomas Fenzl, Cathrine Haller, Wolfgang Guggemos, Jan Hesse, Adinda Harle, Gisela Bretzel, Stefan Sack, Clemens Wendtner, Thomas Löscher, Nicole Berens-Riha

**Affiliations:** Department of Haematology, Oncology, Immunology, Palliative Care, Infectious Diseases and Tropical Medicine, Klinikum Schwabing, Akademisches Lehrkrankenhaus der Ludwig-Maximilians-Universität, Kölner Platz 1, 80804 Munich, Germany; Department of Cardiology, Pneumology, and Intensive Care Medicine, Klinikum Schwabing, Akademisches Lehrkrankenhaus der Ludwig-Maximilians-Universität, Kölner Platz 1, 80804 Munich, Germany; Department of Infectious Diseases and Tropical Medicine (DITM), University Hospital, Ludwig-Maximilians-University, Leopoldstrasse 5, 80802 Munich, Germany

**Keywords:** *Plasmodium knowlesi*, Imported malaria, Severe malaria, Multi-organ failure

## Abstract

During the last two decades human infections with *Plasmodium knowlesi* are increasingly diagnosed in South East Asia and have also been reported in travellers. A severe case of imported *P. knowlesi* infection in a 73-year old German is presented, who had been travelling through Myanmar and Thailand for three weeks. Microscopy showed a parasitaemia of 3% and different parasite stages including band-forms resembling *Plasmodium malariae*. Due to the clinical picture of severe malaria and the microscopical aspect (combination of parasites resembling *P. malariae* and *Plasmodium falciparum*), *P. knowlesi* was suspected. The patient was treated with intravenous quinine; he was put on mechanical ventilation and catecholamines due to cardiorespiratory failure. Parasitaemia was cleared rapidly but renal function deteriorated resulting in intermittent haemodialysis. The patient was hospitalized for six weeks but he recovered completely without any physical sequelae. *Plasmodium knowlesi* mono-infection was confirmed by molecular methods later on.

*Plasmodium knowlesi* infection has to be taken into account in feverish travellers returning from Thailand/Myanmar. Moreover this species can cause life-threatening or even lethal complications. Accordingly severe *P. knowlesi* infection should be treated like severe *P. falciparum* infections.

## Background

A severe case of *Plasmodium knowlesi* infection in a 73-year old man who had been travelling three weeks through Burma and southern Thailand in November/December 2013 is described.

According to published cases, this is the third imported infection with *P. knowlesi* to Germany and the most severe described so far. The severe course of the disease was likely due to delayed medical presentation by the patient and thus diagnosis could only be established seven days after onset of symptoms.

*Plasmodium knowlesi* is known since the early 1930, it affects macaques endemic in Southeast Asia and it has been named in honour of Robert Knowles (1883-1936; School of Tropical Medicine and Hygiene/Calcutta). Knowles first described this *Plasmodium* species in 1930 and he even showed the capacity of artificial human infection with subsequent malaria symptoms. During the last two decades, human *P. knowlesi* cases are found more and more often in many countries in Southeast Asia.

## Case presentation

The 73-year old German patient had been travelling in December 2013 for three weeks for diving and rainforest excursions; he visited the island of Mecleod (Myanmar) and in Thailand Khura Buri, the island Koh Ra, Khao Lak, and Phuket. The day after his return, he developed high fever and chills. He had not taken anti-malarial chemoprophylaxis. After five days with high fever at home (six days after returning from Southeast-Asia), the patient presented to his general practitioner who referred him immediately to the nearest hospital.

On admission, the patient was already hypotonic and in respiratory distress with signs of somnolence (GSCS 7-8, RR 70/50 mmHg, pulse 115/min respiratory frequency 18-20/min, temperature 39,4°C, SP0_2_ 74%). Due to symptoms of shock, decreased gas exchange (pH 7,28, HC03 18 mmol/l) and pulmonary distress (tachypnoea), the patient was put on mechanical ventilation. Catecholamines had to be administered to maintain sufficient perfusion and circulation. Initially, sepsis due to a urinary tract infection was suspected and antibiotic therapy with ciprofloxacin was initialized. However, the patient’s condition deteriorated rapidly and piperacillin/sulbactam were added to the antibiotic regimen. As the thrombocyte count dropped dramatically (30,000/nl), malaria infection was suspected. The Department of Infectious Diseases and Tropical Medicine at Schwabing Hospital in Munich was contacted and the patient was transferred to its ICU via helicopter.

On a moderate dosage of catecholamines (0.4 μg arterenol/min), vital parameters were stable. Laboratory parameters are displayed in Tables 1 and 2. Ventilation was performed with 50% oxygen and a PEEP of 8 cm H_2_O in BIPAP modus. Abdominal ultrasound showed a slight splenomegaly (15 cm diameter at the hilus) and small bilateral pleural effusions. The chest x-ray at the first day showed beginning ARDS (interstitial opacities, small pleural effusions and congestion), chest x-ray four days later showed bilateral basal infiltrations.

**Table 1:** **Important laboratory parameters**

Parameter	Creatinine	GFR	Procalcitonin	CRP	LDH	Bilirubin
(normal range)	(0.7-1.2 mg/dL)	(>60 mL/min)	(<0.5 μg/L)	(<5 mg/L)	(125-220 U/L)	(0,2-1,2 mg/dL)
Day 1	**1.7**	**40**	**32.4**	**300.2**	415	**1.4**
Day 2	**3.1**	**19**	**38.7**	**342.6**	586	**2.2**
Day 5	**5.1 (HD)**	**11**	**8.2**	**149**	924	**1.5**
Day 30	**1.9**	**32**	0,1	5.8	391	0.8

Bold means pathological values.

**Table 2:** **Blood count over time**

Parameter	Erythrocyte count	Haemoglobin	Thrombocyte count	Leucocyte count
(normal range)	(4.5-5.9/pL)	(13.5-17 g/dL)	(140-360/nL)	(3.5-9.8/nL)
Day 1	**3.7**	**11.3**	**39**	8.8
Day 2	**3.7**	**10.9**	**86**	**11.1**
Day 5	**2.7**	**8.0**	153	**25.2**
Day 30	**3.2**	**9.8**	391	5.7

Bold means pathological values.

**Figure 1:**
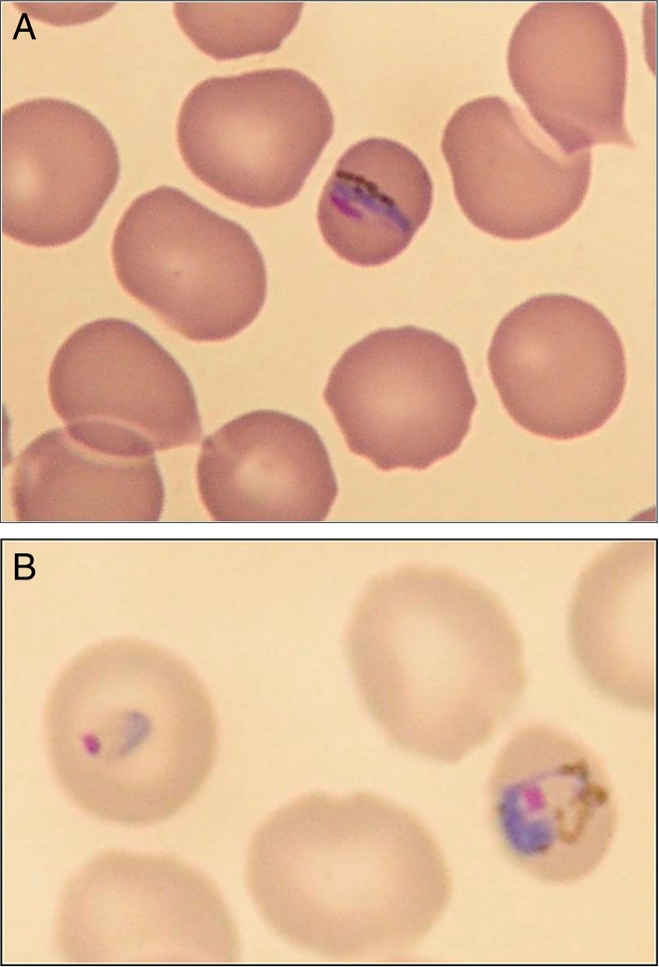
**Microscopic aspect of Plasmodium knowlesi in a blood smear of the presented case prior to initiation of treatment with quinine. (A)** Band form late trophozoite; **(B)** Early trophozoite or ring form (left) and late trophozoite (right).

Thin blood smears revealed 3% parasitaemia. The trophozoites resembled *Plasmodium falciparum* and *Plasmodium malariae*. Band-forms and gametocytes reminded of *P. malariae* (Figure 1)*.*

*Plasmodium knowlesi* infection was, therefore, suspected and therapy started with intravenous quinine (due to the lack of artesunate at the time of diagnosis) in combination with doxycycline (200 mg per day) immediately upon arrival at the intensive care unit (ICU) of Schwabing hospital. The patient showed a quick response to quinine and was completely cleared from parasites within 48 hours. A subsequently developed ventilator-associated pneumonia was treated successfully with meropenem. Creatinine and glomerular filtration rate (GFR) were already impaired on admission and acute anuric kidney failure developed within the first day (Table 1). The patient needed haemodialysis for five weeks but the kidney function finally recovered and the patient could be discharged without sequelae.

The severe course of the disease with malaria-related lung and kidney failure was likely due to delayed medical presentation by the patient and thus diagnosis could only be established seven days after onset of symptoms.

Definite diagnosis was confirmed by PCR (DITM). A *Plasmodium* genus-specific real-time PCR was positive but the multiplex PCR for the other four human *Plasmodium* species was negative (FTD Malaria, Mikrogen Diagnostik, Neuried, Germany). Two *P. knowlesi*-specific nested PCRs based on the 18S rRNA gene showed the expected amplicons [1, 2]. Direct DNA sequencing of PCR products confirmed 99% nucleotide concordance with *P. knowlesi* strains from Thailand and Malaysia.

Written informed consent of the patient was procured for publication.

## Discussion

*Plasmodium knowlesi* is known since the early 1930 as a parasite of macaques endemic in Southeast Asia [3]. In 1965, naturally acquired human infection was first reported in an American tourist travelling through Malaysia [4]. Retrospectively, Singh *et al*. recently detected a high prevalence of *P. knowlesi* in humans in East Malaysia but it still seems to be under-diagnosed due to diagnostic constraints and possibly also due to a lack of awareness concerning this *Plasmodium* species [2, 5–7].

In certain areas of Malaysia (Sarawak, Borneo), the prevalence of human *P. knowlesi* was as high as 50%, whereas in Thailand it was around 1% [1, 8–11]. Human *P. knowlesi* infections were observed so far in Malaysia, Thailand, Burma, Vietnam, the Philippines, Singapore, and Indonesia [12]. As a travel-related disease it was still rarely described [5, 6, 12]. This is the third documented case in Germany and the number of infections in other non-endemic countries is likewise low [4, 5, 11].

Along with better diagnostic tools and awareness raising, an increase of *P. knowlesi* infections in humans during the last two decades is discussed and might be attributable to a reduction of the natural habitat of macaques, the invasion of humans into rainforests (e g, for economical reasons) and an increasing number of the *Anopheles* vectors feeding on both humans and monkeys [11].

The main vectors for *P. knowlesi*, *Anopheles cracens* and *Anopheles lateens,* feed on macaques as well as on humans [11]. Also, the predominantly antropophilic *Anopheles stephensi* which is widely distributed in Southeast Asia, can be easily infected with *P. knowlesi*, and were found in rural as well as urban areas [13]. Most reported cases of *P. knowlesi* had visited rainforest regions prior to manifestation of malaria [14]. So far only one case reported in the medical literature describes a *P. knowlesi* infection without prior rainforest excursions [14]. This indicates a direct link to the natural habitat of *P. knowlesi.* So far, there is no evidence of a stable human-*Anopheles*-human transmission [6]. The patient presented in this report most probably contracted the infection in southern Thailand. Ranong province was also the probable site of infection in two other German *P. knowlesi* cases published so far [15, 16].

*Plasmodium knowlesi* may take a severe, even fatal course, such as described for *P. falciparum infections,* in about 7%, as *P. knowlesi* can lead to hyperparasitaemia [5, 17]. Like *P. falciparum*, *P. knowlesi* can invade all erythrocytes, not only very young erythrocytes as in *Plasmodium vivax* and *Plasmodium ovale,* or very mature erythrocytes as in *P. malariae* [5]. Moreover, the intra-erythrocytic cycle lasts only 24 hours, which is much shorter than in any other human malaria species and this can lead to a rapid progression of *P. knowlesi* infection [5, 13].

Therefore, immediate initiation of treatment and vigilant clinical observation is essential. As *P. knowlesi* has no liver hypnozoite stage the infection does not relapse [5]. Nevertheless, there are cases of complicated malaria due to *P. knowlesi* with renal failure and respiratory distress in patients with low parasitaemia [15]. On the other hand, data indicate asymptomatic infection as well [1, 6].

The presented patient faced a severe, life-threatening course of the disease with 3% parasitaemia.

He developed complete anuric kidney failure, impaired consciousness, respiratory distress and circulatory deficits, and therefore needed mechanical ventilation and catecholamines shortly after hospital admission. In this case, initial suspicion of a *P. knowlesi* infection was based on the detection of intra-erythrocytic ring forms which resembled *P. falciparum* as well as band forms and schizonts indistinguishable from *P. malariae* in the patient’s thin blood smear. This is a typical aspect of *P. knowlesi* infection and, therefore, *P. knowlesi* is prone to misidentification and needs further confirmation by means of PCR [1, 6].

The laboratory results often reveal a significant to severe thrombocytopaenia, signs of haemolysis with anaemia, and elevated liver enzymes, like as in this patient. A close correlation between *P. knowlesi* parasitaemia and platelet count is assumed and it seems that the degree of the thrombocytopaenia could be even worse than in other types of malaria [8]. However, it usually quickly improves with successful parasite clearance [18, 19].

Other important signs of a life-threatening situation in a *P. knowlesi* infection are: acidosis, rapid drop in erythrocyte count, jaundice, renal impairment or even renal failure, respiratory distress, and hypotonia [5, 18, 20]. Patients with impaired consciousness and decreased Glasgow Coma Scores have been described in severe and fatal cases but other signs and symptoms such as unrousable coma did not satisfy the WHO criteria for cerebral malaria [20]. Pathological brain alterations in a fatal case of human *P. knowlesi* malaria were similar to those found in *P. falciparum* malaria with multiple petechial haemorrhages and sequestration of pigmented parasitized red blood cells in capillaries and venules. However, unlike in falciparum malaria ICAM 1 (intracellular-adhesion-molecule) was not found to be up-regulated, therefore the pathophysiological aspects seem to be different [21].

Undoubtedly the patient suffered from severe malaria corresponding with the WHO classification and his altered mental status (GSCS 7-8) suggests cerebral involvement. The patient had anuric kidney and lung failure, severe haemolysis and cerebral impairment qualifying him for severe malaria with multiorgan failure (apart from the hyperparasitaemia).

Autoptic changes in the lung tissue of a patient who had died from *P. knowlesi* malaria suggest that lung injury occurs in non-falciparum-malaria and ARDS has to be taken into account [21].

Moreover a case series of five patients who died from knowlesi malaria in Malaysia showed that pulmonary problems and respiratory distress were quite common in this infection [22].

So far there is no standard treatment protocol for *P. knowlesi* malaria based on RCTs but it seems that most of the anti-malarial drugs are effective [6, 13]. However, one case report showed an increase in parasitaemia after application of mefloquine as a possible hint for resistance [13]. Both parenteral quinine and artemisinins have been effective in the treatment of severe *P. knowlesi* malaria [20]. Current WHO guidelines recommend artesunate i.v. as a first choice in treating severe malaria caused by all plasmodium species as the clearance of parasites is rapid with less side-effects [8, 20, 22–24]. Since parenteral artemisinin were not readily available, the patient presented was treated with quinine per infusionem and showed a rapid clearance of parasitaemia.

## Conclusion

Infection due to *P. knowlesi* has to be taken into account in feverish patients returning from Southeast Asia (namely from Malaysia, Borneo and Ranong Province in Thailand), especially when the parasites in malaria blood slides have close resemblance to *P. malariae* and/or *P. falciparum*. PCR diagnosis is crucial for definite characterization of *P. knowlesi* as microscopic differentiation is hardly possible. As a travel related disease *P. knowlesi* malaria is still highly exceptional with only a small number of cases reported every year from non-endemic, western countries. *Plasmodium knowlesi* may cause a rapid clinical deterioration as the intra-erythrocytic cycle is only 24 hours and in almost 7% it has a severe, life-threatening course with multi-organ failure (such as in *P. falciparum* malaria). Correspondingly, severe *P. knowlesi* malaria should be treated like severe *P. falciparum* malaria.
